# Perillaldehyde improves cognitive function in vivo and in vitro by inhibiting neuronal damage via blocking TRPM2/NMDAR pathway

**DOI:** 10.1186/s13020-021-00545-9

**Published:** 2021-12-13

**Authors:** Yue Qiu, Xian-jun Xue, Geng Liu, Miao-miao Shen, Chun-yan Chao, Jie Zhang, Ya-qi Guo, Qian-qian Niu, Ya-nan Yu, Yu-ting Song, Huan-huan Wang, Shuang-xi Wang, Yu-jing Chen, Lin-hua Jiang, Peng Li, Ya-ling Yin

**Affiliations:** 1 College of Pharmacy, Henan International Joint Laboratory of Cardiovascular Remodeling and Drug Intervention, Xinxiang Key Laboratory of Vascular Remodeling Intervention and Molecular Targeted Therapy Drug Development, Xinxiang Medical University, Xinxiang, 453003 China; 2 Sino-UK Joint Laboratory of Brain Function and Injury and Department of Physiology and Neurobiology, Department of Physiology and Pathophysiology, School of Basic Medical Sciences, Xinxiang Medical University, Xinxiang, 453003 China; 3 School of Biomedical Sciences, Faculty of Biological Sciences, University of Leeds, Leeds, UK; 4 Puyang Oilfield General Hospital, Puyang, 457001 China; 5 The First Affiliated Hospital, Xinxiang Medical University, Xinxiang, 453003 China; 6 The People’s Hospital of Anyang City, Anyang, 455001 China; 7 Huang Huai University, Zhumadian, 463000 China

**Keywords:** Vascular dementia, Perillaldehyde, TRPM2, NMDAR pathway

## Abstract

**Background:**

Vascular cognitive dysfunction in patients with vascular dementia (VD) is a kind of severe cognitive dysfunction syndrome caused by cerebrovascular diseases. At present, effective drugs to improve the cognitive function of VD patients still need to be explored. Transient Receptor Potential Melastatin 2 (TRPM2) channel is a nonspecific cation channel that plays a key role in the toxic death of neurons. Perillaldehyde (PAE) has the protective effect of epilepsy and insomnia and other central nervous system diseases. The aim of this study is to explore whether PAE improves cognitive function in VD rats and to investigate the potential mechanisms in vivo and vitro.

**Methods:**

VD rats were induced by bilateral common carotid arteries occlusion (2-vessel occlusion [2VO]) and treated with PAE for 4 weeks. The neuroprotective effects of PAE was subsequently assessed by the Morris water maze, hematoxylin–eosin (HE) staining, Golgi staining, electron microscopy, Neuron-specific nuclear protein (Neu N) staining, and TdT-mediated dUTP nick end labeling (TUNEL) staining. After primary hippocampal neurons were isolated, cell viability was detected by MTT assay and intracellular Ca^2+^ concentration was detected by calcium imaging assay. The content of Nitriteoxide (NO), Malondialdehyde (MDA) and Superoxide dismutase (SOD) activity in serum of rats were observed by Enzyme Linked Immunosorbent Assay (ELISA). Immunohistochemistry, Western blot, and Confocal laser scanning were used to detect the expression levels of *N*-methyl-d-asprtate receptor-2B (NR2B) and TRPM2.

**Results:**

The results showed that PAE can improve the number and activity of neurons, increase the length and number of dendrites in hippocampus, decrease the Vv value and PE value of neuronal nucleus and mitochondrial structure significantly, increase the s value and L value in nucleus structure, decrease the s value and L value in mitochondrial structure, and improve the learning and memory ability of rats significantly. And PAE can strengthen the ability of antioxidant stress confirmed by increasing the activity of SOD and reducing the production of MDA. The results of western blot, immunohistochemistry and immunofluorescence showed that PAE could reduce the level of TRPM2 and increase the expression of NR2B.

**Conclusions:**

Taken together, our findings provide evidence that the neuroprotective effects of PAE in VD rats maybe through TRPM2 inhibition and subsequent activation of NMDAR signaling pathway.

## Introduction

Vascular dementia is a clinical dementia syndrome caused by brain tissue damage directly or indirectly caused by various vascular injury factors, which is often characterized by impaired memory and cognitive function or a group of neurocognitive disorders with decreased language function and/or emotional and personality disorders as the main clinical manifestations [1–4]. At present, The prevalence of VD in China is 1.1–3.0%, and the annual incidence rate is 5–9/1000 [5]. Therefore, research on finding new drugs improve vascular cognitive dysfunction with fewer side effects and better efficacy is continuing.

In the prescription of traditional Chinese medicine, there are kinds of traditional Chinese medicine with the function of central nervous system protective effects, for example milkvetch root, san qi and Szechwan lovage rhizome, et al. [6]. Monoterpene Perillaldehyde (PAE) is a major component of the essential oil extracted from perilla plants [7, 8]. In recent years, PAE has achieved much attention because of its antifungal and other microbial activities [9]. In addition, a study has revealed that PAE has good antioxidant activity [10]. Therefore, more pharmacological actions and mechanisms of PAE need to be explored.

*N*-Methyl-d-aspartate receptor (NMDAR/NR) is a ligand-gated ion channel, including NR1, NR2BA, NR2BB and other subtypes, which plays a key role in synaptic protein shaping, axonal structure development, neural circuit formation, learning and memory of neurons [11, 12]. NMDAR is a key component of aspartate excitotoxicity. NMDAR postsynaptic membrane signal transduction pathway is involved in cognitive dysfunction. Inhibition or overexcitation of NMDAR can cause neuronal dysfunction. CaMK II and PSD95 are the main functional proteins in this pathway [13, 14]. Transient receptor potential melastatin 2 (TRPM2) channel has many physiological functions, including inflammatory response, insulin production and synaptic plasticity [15]. In the glutamate-independent Ca^2+^ mechanism of ischemic neuronal injury, TRPM2 is involved in mediating the increase of toxic Ca^2+^ influx and aggravating neuronal injury [16]. It is reported that H_2_O_2_-induced activation of TRPM2 channels can lead to the influx of Ca^2+^ in cortical and hippocampal neurons, suggesting that TRPM2 may mediate its effect on neuronal death through oxidation [17]. Therefore, up-regulation of NMDAR receptor expression and down-regulation of TRPM2 expression to reduce excessive Ca^2+^ influx may be a neuroprotective mechanism. In this study, we aimed to examine the effects of PAE in VD rats, and found that PAE improved the morphology of neurons and prevented cognitive dysfunction in VD rats by inhibiting the expression of TRPM2 and activating NMDAR pathway.

## Methods and materials

### Major reagent

Perillaldehyde essential oil (PAEEO) was obtained by Sigma-Aldrich (St. Louis, MO). Primary antibodies including NR2B, TRPM2, β-actin were purchased from Santa Cruz Company. Mouse neuron growth factor (mNGF) was purchased from the First Affiliated Hospital of Xinxiang Medical University (Xinxiang, China) and prepared in saline consisting of 1% Tween 80.

### Animals

Male Wistar rats weighing 260–270 g were supplied by the Experimental Animal Center of Henan Province (license number: SYXK-Yu-005-0012). All rats were housed individually in cages and given free access to food and water. The living temperature and humidity range was 25 ± 1 °C and 55% ± 5% with a 12 h light/dark cycle. The study was maintained in accordance with the guidelines outlined by the National Institutes of Health Guide for the Care and Use of Laboratory. The experiment protocol was reviewed and approved by the Animal Care and use Committee of the Xinxiang Medical University.

### Permanent common carotid artery occlusion

VD rats were established by bilateral common carotid arteries occlusion (2-vessel occlusion [2VO]). Rats were weighed and intraperitoneally injected with 30 mg/kg pentobarbital sodium for anesthesia. The bilateral common arteries were exposed and separated from the vagus nerve, then ligated with 5–0 silk thread. The sham operation group was operated in the same way, but it was not arterial ligated. During the whole operation, the operation should be as mild as possible to reduce the pain of animals.

### Primary culture of hippocampal neurons

After pregnant rats were anesthetized, the abdomen of pregnant rats were disinfected with 75% alcohol, and then the fetal rats were carefully taken out. The fetal rats were immersed in 0.9% normal saline and 75% alcohol successively at 4 °C for 1 min each. The fetal rats were decapitated, and the heads were immediately immersed in artificial cerebrospinal fluid at 0 °C for 10 s. The surgical scissors kept parallel to the decapitation surface of rats. The olfactory bulb end of the transverse plane of the rat skull was cut horizontally, and then the longitudinal cutting was carried out along the coronal plane of both sides of the rat skull. After cutting the rat skull, the head of the olfactory bulb end was clamped firmly with surgical tweezers, and the whole skull was lifted up to remove the rat brain Hemispheres were immediately immersed in artificial cerebrospinal fluid at 0 °C for 2 min. Under the stereomicroscope, the hippocampal tissue was quickly separated with ophthalmic scissors and fine tweezers, and quickly transferred to the culture dish of 5 mL balanced salt solution on the ice bag.

The 0.5 mm × 0.5 mm square hippocampal tissue blocks were cut with ophthalmic scissors, and 0.25% trypsin was added into each hippocampal tissue block until the final concentration of trypsin was 0.125%. Put the culture dish into the cell incubator for incubation, take out the culture dish every 5 min and gently shake it to make sure that the hippocampal tissue is evenly distributed in the culture dish. After 15 min, 1 mL fetal bovine serum was added to the culture dish and mixed with pipette. Trypsin digestion was stopped and the supernatant was sucked away. 3 mL of culture medium was added to the culture dish, and the cells were repeatedly blown with the gun head 10 times. In the process of operation, the suspension should be slowly sucked and blown at a uniform speed. The cell suspension was seeded in a poly lysine coated cell culture plate or dish for culture.

### Experimental scheme in vivo

The essential oil was first dissolved in DMSO at the concentration of 10 g/mL, and then suspended in saline containing 1% Tween80 to prepare for three concentrations of low, medium, and high doses of 50 mg/kg, 100 mg/kg, and 150 mg/kg for rats daily drinking after 2VO surgical operation. Mouse neuron growth factor (mNGF) was administered by a single intraperitoneal injection. All rats were randomly divided into eight groups of six animals. Group 1: Saline group (Sham); Group 2: Vascular dementia group (Model); Group 3: Perillaldehyde group (Sham + PAE-150 mg/kg); Group 4: Vascular dementia + Perillaldehyde (50 mg/kg) group (PAE-50 mg/kg); Group 5: Vascular dementia + Perillaldehyde (100 mg/kg) group (PAE-100 mg/kg); Group 6: Vascular dementia + Perillaldehyde (150 mg/kg) group (PAE-150 mg/kg). Group 7: Vascular dementia + mNGF (20 mg/kg) group (mNGF). Group 8: Vascular dementia + Perillaldehyde (150 mg/kg) + mNGF (20 mg/kg) group (PAE-mNGF). Before sacrifice, the learning and memory behavior of rats were assessed. The hippocampus of rats was isolated for morphological analysis.

### Morris water maze (MWM) test

The Morris water maze test (MWM) was used for memory and learning behavior assessment [18]. On the 1st to 4th day of the experiment, rats were put into the water maze with their heads facing the pool wall in the order of East, West, South and North. Each experiment was limited to 60 s. If the rats found the platform within 60 s after entering the water, they were allowed to stay on the platform for 10 s; if the rats could not find the platform within 60 s after entering the water, the experimenters placed the rats on the platform and stayed for 10 s. Each rat was trained 4 times a day with an interval of 20 min. On the 5th day of the experiment, the platform was removed, and the rats were put into any water entry point in the water maze. The swimming track of rats within 1 min was recorded by a video recording system (Columbus Instruments, USA). Swim distance in quadrant (m), Swim distance out quadrant (m), Swim distance (m), Swim distance in quadrant/Swim distance, Swim time in quadrant (s), Swim time out quadrant (s), Swim time in quadrant/Swim time, Swim speed in quadrant (mm/s), Swim speed out quadrant (mm/s), Average swim speed (mm/s), Number of escape were analyzed by image pro plus medical image processing system.

### HE staining

The rats of each group were anesthetized with chloral hydrate (350 mg/kg, ip), and then perfused with PBS and 4% paraformaldehyde. The whole brain was removed and then post-fixed in 4% paraformaldehyde. After regular perfusion and fixation, the brain segments containing hippocampus were obtained and paraffin embedded. The frontal slices were cut continuously with a thickness of 5 μm using a microtome (Leica RM2135, Nussloch, Germany). The sections were stained with hematoxylin and eosin and collected pictures with digital pathological section Scanner (3DHISTECH, Pannoramic SCAN, EU). HE staining was carried out to detect the global morphology of tissues as described previously [19].

### Golgi staining

The hippocampal tissue was cut into 80 μm thick slices by cryosectioning machine at − 20 to − 22 °C. According to the instructions, the sections were stained with Golgi staining kit (Hito Golgi-Cox Optimstain kit, Hitobiotec, USA). After the sections were sealed with neutral gum, they were dried at room temperature. The number of dendritic spines and morphology of neurons were observed. Under the microscope, 5 neurons were located in each group, and 3 segments of dendrites with grade 2–3 branches were selected respectively. The measurement range of 100 μm length was marked, and the number of dendritic spines was counted.

### Electron microscope quantitative analysis

Each group of specimens took 30 cell fields, each field took a more appropriate range, unity square large multiple was 1000 times for ultrastructural observation and photography. Using SIS electron microscope image analysis software to test, under the interactive operation mode, the mitochondria and nuclei of each picture are respectively traced by the cursor, and the primary parameters such as perimeter, area, roundness, long axis, short axis, as well as the area of each picture are tested. According to the principle of miss's, the formula VX/VR = ax/AR (i.e. the ratio of cross-sectional area of two structures on two-dimensional section image, etc.) is derived. According to its volume ratio in three-dimensional space, the relevant secondary parameters volume density (Vv), shape factor (PE), average area (s) and average perimeter (L) are calculated. The thickness of presynaptic membrane and the width of postsynaptic space were measured.

### TUNEL assay

TUNEL is a common method for detecting DNA fragmentation that results from apoptosis signaling cascades. One Step TUNEL Apoptosis Assay Kit was purchased from Beyotime Biotechnology and operated according to the instructions. All the images were collected in the digital pathological section scanner (3D HISTECH, Pannoramic SCAN, EU).

### Western Blotting

Western blot was used to detect the expression of TRPM2 and NR2B in hippocampus tissues. After anesthesia, the hippocampal tissues were taken out and homogenized on in RIPA buffer with 1 mM PMSF. The proteins were quantified by BCA Protein Assay kit (Thermo Scientific), separated by SDS-PAGE, transferred to PVDF membrane and detected by specific antibody. Band intensity was measured by densitometry (model GS-700, Imaging Densitometer; Bio-Rad). The ratio of β-actin to NR2B and TRPM2 was used to represent the expression of NR2B and TRPM2 in hippocampus respectively.

### Immunohistochemistry and confocal laser scanning

For immunohistochemistry staining, the hippocampal tissue slices were frozen and sectioned with a cryostat microtome (8 µm). The sections were incubated for 10 min for antigen recovery, and then blocked with 5% BSA for 30 min. Brain sections were incubated with a primary antibody overnight at 4 °C to detect the expression of TRPM2 and NR2B respectively. Then incubated with secondary antibodies for 1 h at room temperature and detected with DAB. For immunofluorescence staining, brain sections and cells were blocked with 5% BSA and then incubated with primary antibodies overnight at 4 °C to detect the expression of neurons (Neu N), the colocalization between TRPM2 and NR2B respectively. Subsequently, the sections and cells were washed three times with PBST and incubated with secondary fluorescent antibody in the dark for 2 h at room temperature and tested after mounting with DAPI. All the images were collected in the digital pathological section scanner (3D HISTECH, Pannoramic SCAN, EU).

### Fluorescence Ca^2+^ concentration detection

The Fluo3-AM calcium ion fluorescent probe (Ringer’s) was used to detect Ca^2+^ concentration. The signal sampling rate was adjusted to 1 Hz with a 488 nm argon laser. Most cells were delineated as the region of interest (ROI). The time series imaging of ZEN2009 software was used to observe spontaneous calcium shock imaging in the resting state of the neurons under LSM 510 system laser confocal microscope. Denoted by F/F_0_ Spontaneous calcium oscillations of neurons. F represents the relative fluorescence signal value of neuron cells, and F_0_ represents the average fluorescence signal value recorded within 2 min. F, F_max_ and F_min_ collected by LSM 510 system laser confocal microscope, enter the following formula:

$$\left[ {{\text{Ca}}^{2 + } } \right]_{{\text{i}}} = \left[ {\left( {{\text{F}} - {\text{F}}_{\min } } \right)/\left( {{\text{F}}_{\max } - {\text{F}}} \right)} \right] \times {\text{K}}_{{\text{d}}} \times {\text{S}}{.}$$

Among them, K_d_ is 224 mM; F_min_ is the F value measured after the buffer lysed cells complexed with free calcium ions. The F value of the buffer solution contained 0.05% TritonX-100, 6.6 mM EGAT and 40 mM Tris–HCl buffer without Ca^2+^; the F value measured after the buffer solution with calcium ion concentration of 0.05% TritonX-100 is 2 mM. S is the ratio of the minimum fluorescence intensity to the maximum fluorescence intensity measured at 380 nm excitation wavelength.

### MTT staining

Hippocampal tissue of rats was isolated and cultured for primary neurons. Cells in the logarithmic phase of the cell cycle were collected and cultured on cell plate. Thus, the 20 μL MTS solution was added to each well and continued to incubate for 1–2 h. Then the cell plate was put into the dtx880 multi-functional microplate reader, at the wavelength range of 490 nm to measure the light absorption value of each well. Finally, the cell growth curve with time and absorption value as abscissa and ordinate respectively was drawn to calculate the cell activity.

### ELISA analysis

Animals were sacrificed and brains were quickly harvested. Then, the hippocampus was extracted by using a dissecting microscope and weighted and homogenized in liquid nitrogen. The content of nitric oxide (NO), malondialdehyde (MDA) and the activity of superoxide dismutase (SOD) were detected by ELISA according to the manufacturer’s instructions (FineTest, China).

### Statistical analysis

Data are presented as the mean ± SEM. Raw data was statistically analyzed with Graph Pad Prism 5.0. MWM test escape latencies and swimming speeds were analyzed using two-way analysis of variance (ANOVA) with repeated measures. The other data was analyzed using one-way ANOVA. Fisher’s least-significant difference post hoc test was used to test the differences between two groups. The value of P < 0.05 was considered statistically significant.

## Results

### PAE significantly attenuated spatial cognitive impairment in VD rats

**Fig. 1:**
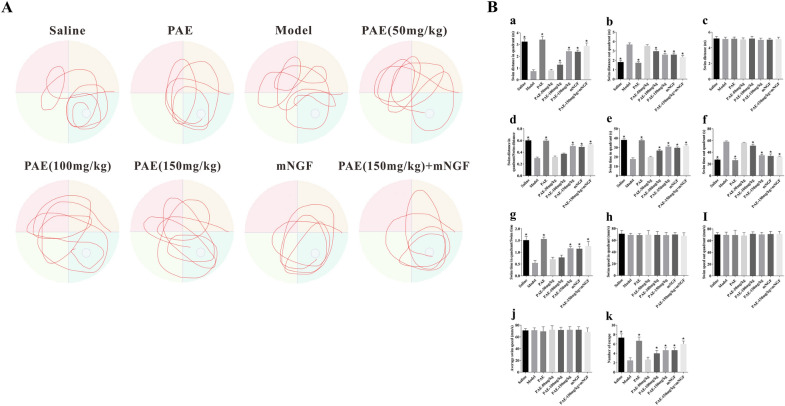
Effects of PAE on spatial learning and memory. **A** The trajectory of rats in each group reaching the platform in the swimming pool. **B** Animals of PAE-treated group significantly increased Swim distance in quadrant, Swim distance in quadrant/Swim distance, Swim time in quadrant, Swim time in quadrant/Swim time, Number of escape than other groups (**a**, **d**, **e**, **g**, **k**). Animals of PAE-treated group significantly reduced Swim distance out quadrant, Swim distance out quadrant than other groups (**b**, **f**). PAE did not affect the locomotor ability of animals and all animals showed swimming abilities during habituation (**c**, **h**, **i**, **j**). Data are reported as mean ± SEM. N is 6 in each group. *P < 0.05 vs. Model group. All results were analysed using a one-way ANOVA followed by Newman-Student’s t-test

We first performed the Morris water maze test (MWM) for spatial learning and memory behavior assessment. The trajectory of rats in each group reaching the platform in the swimming pool was shown in Fig. 1A. And the statistical results shown as in Fig. 1B, swimming distance in quadrant, ratio of swimming distance in quadrant to out of quadrant, swimming time in quadrant, swimming time in quadrant to total swimming time and number of escapes were increased in PAE-treated rats compared with VD rats (Fig. 1B, a, d, e, g, k, P < 0.05). These findings suggest that the learning and memory ability of the VD rats has significantly decreased. After PAE treatment (100 mg/kg, 150 mg/kg), the learning and memory abilities of rats were significantly improved, and there was a significant difference compared with the model group (P < 0.05). When high dose PAE and mouse nerve growth factor (mNGF) intervened at the same time, the improvement effect of PAE on vascular cognitive impairment was significantly enhanced, and there was a difference compared with mNGF intervention group. Conversely, there was no significant difference in total swimming distance and swimming speed (Fig. 1B, c, h, i, j, P < 0.05), demonstrating that motor function was not impaired by PAE.

### PAE significantly improved the morphology and structure of hippocampal neurons in VD rats

**Fig. 2:**
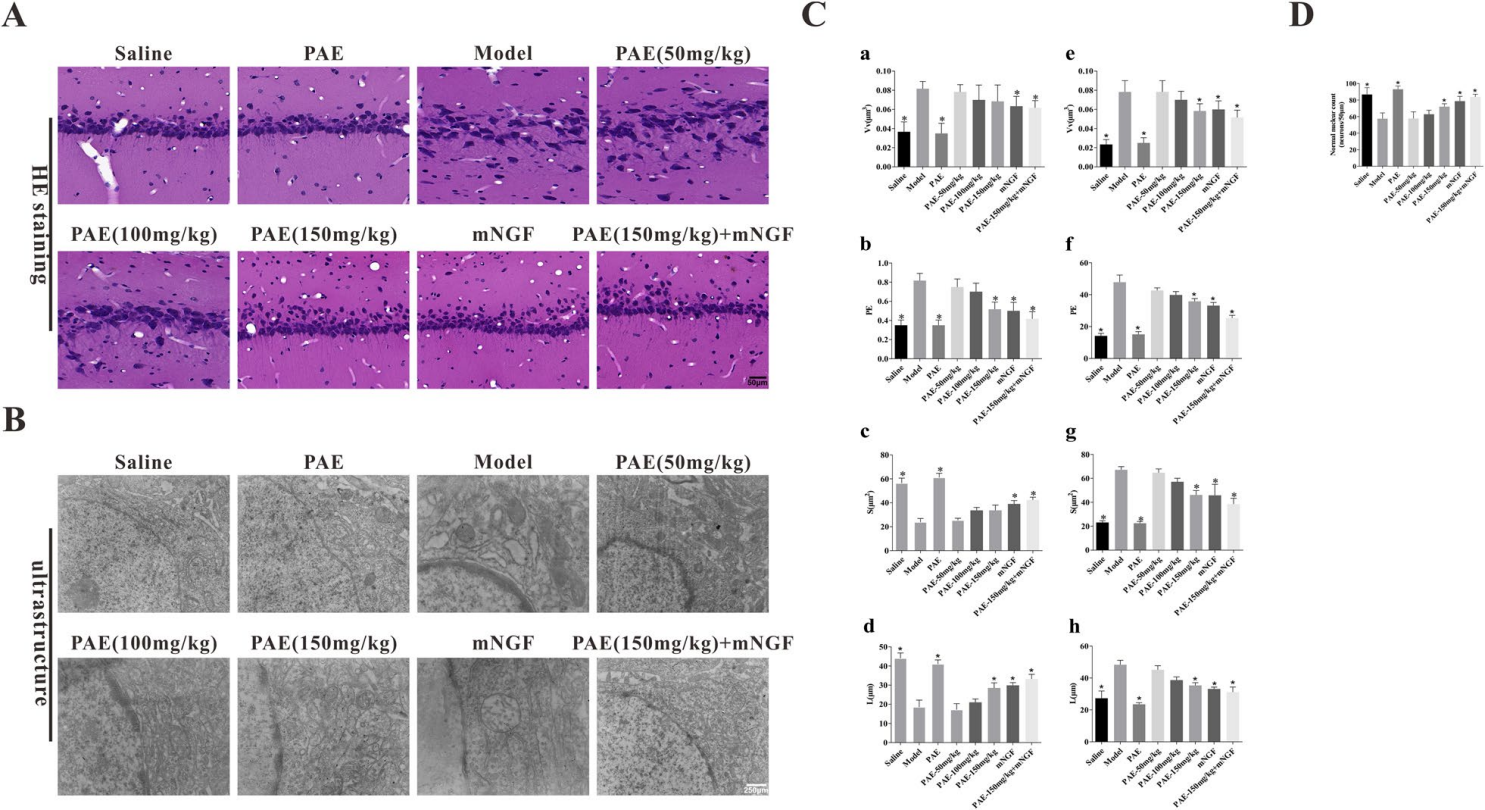
PAE organizes the structure of hippocampus in rats. **A** Morphological analysis was performed under ultrastructure by electron microscope (×400). **B** Morphological analysis was performed under light microscope by hematoxylin and eosin staining (×400). **C** The quantitative analysis of nuclear structure (**C**, **a**–**d**) and mitochondrial structure (**C**, **e**–**h**) in hippocampal CA3 region. All data is expressed as mean ± SEM. N is 6 in each group. *P < 0.05 vs. Model group. All results were analyzed using a one-way ANOVA followed by Newman–Student’s t-test

It is necessary for acquiring spatial information to have an intact hippocampus [20]. HE staining showed that compared with the control group, the model group showed the disordered arrangement of hippocampal neurons and infiltration of inflammatory cells. The pathological changes in the high dose PAE group were alleviated significantly than those in the VD group (Fig. 2A). Further, the high dose of PAE increased the number of normal neuronal nuclei, with significant difference compared with model group (P < 0.05), but no significant difference compared with mNGF control group (P > 0.01) (Fig. 2D). We also tested the morphology of hippocampal neurons by electron microscopy at the end of the experiment. As shown in Fig. 2B, the inner membrane of hippocampal blood–brain barrier in model group was not smooth, the membrane structures were unclear with shrank nucleus and the neurons were smaller and the aggregated chromatin to the edges, the quantitative analysis of nuclear structure in hippocampal CA3 region showed that Vv value and PE value increased, S value and L value decreased (Fig. 2C, a–d), and the quantitative analysis of mitochondrial structure in hippocampal CA3 region showed that the values of Vv, PE, S and L were significantly higher than those in the control group (Fig. 2C, e–h). Treatment of APE can significantly improve the structure of inner membrane and nucleus. When high dose PAE and mNGF intervened at the same time, the improvement effect of PAE on the morphology and activity of hippocampal neurons was significantly enhanced, and the density of pyramidal cells was significantly increased.

### PAE rescued dendritic remodeling of the hippocampal CA3 pyramidal cells in VD rats

**Fig. 3:**
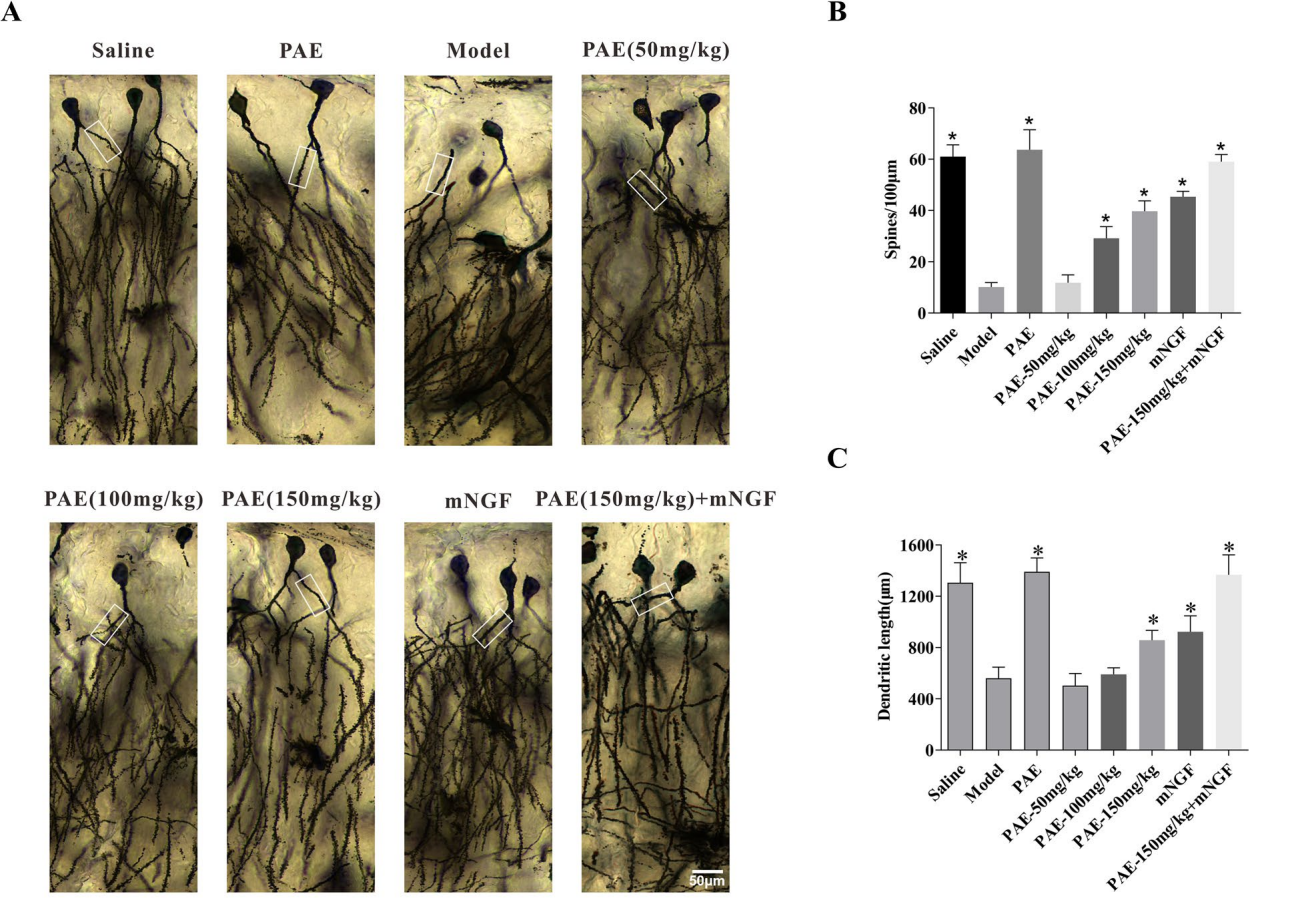
PAE rescues the degeneration of pyramidal neurons in the hippocampus of the VD rats. **A** Golgi-stained neuronal profiles of hippocampus in rats. **B** Trends of changes of spine density and **C** dendritic length in Golgi-stained hippocampus based on Neurolucida-based morphometry. All data is expressed as mean ± SEM. N is 6 in each group. *P < 0.05 vs. Model group. All results were analyzed using a one-way ANOVA followed by Newman–Student’s t-test

The increase and elimination of dendritic spines is interpreted as the acquisition and loss of excitatory synapses, which is considered to be the remodeling of excitatory circuits [21]. Golgi staining can be used to observe the morphology of dendrites. As shown in Fig. 3, the length of dendrites and the number of dendritic spines of hippocampal neurons in the model group were significantly shorter than those in the Saline control group. Treatment of PAE can inhibit the shortening of dendritic length and the number of dendritic spines decrease in hippocampal CA3 neurons induced by 2VO-operated. The number and length of dendritic spines in hippocampal neurons of rats were significantly improved, especially the treatment of high dose PAE could significantly reverse the injury of hippocampal neurons in VD rats. When high dose PAE and mouse nerve growth factor (mNGF) intervened at the same time, the improvement effect of PAE on the number of dendritic spines and dendritic length of hippocampal neurons in VD rats was significantly increased. These findings suggested that PAE could rescue the neuronal dendritic regression induced by 2VO-operated in hippocampal CA3 pyramidal neurons.

### PAE significantly reduced oxidative stress of hippocampus in VD rats

**Fig. 4:**
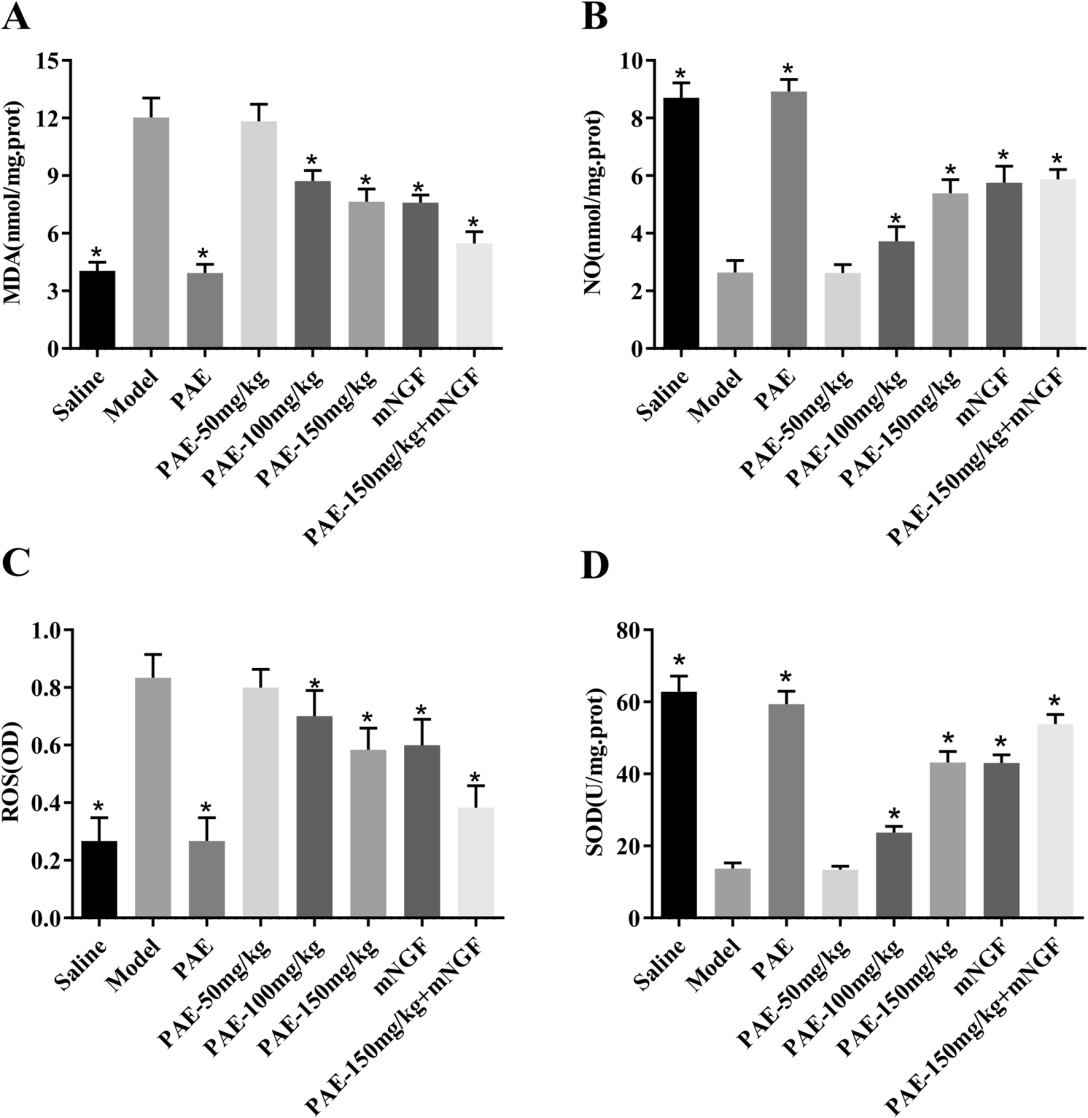
The antioxidant effect of PAE in VD rats. At the end of experiments, rats were killed under anesthesia. Blood was collected to assay serum levels of **A** MDA content by the TBA method, **B** NO production by the Griess method, **C** ROS content by the NBT method, **D** SOD activity by the spectrophotometric method. All data is expressed as mean ± SEM. N is 6 in each group. *P < 0.05 vs. Model group. All results were analyzed using a one-way ANOVA followed by Newman–Student’s t-test

Overproduction of reactive species oxygen (ROS) and inflammation caused by ischemia are two main factors resulting from the pathogenesis of VD. Superoxide dismutase can convert no into ONOO^−^ and further reduce the content of NO, resulting in the occurrence of ischemic cardiovascular disease. Some studies have shown that reducing oxidative stress injury has a neuroprotective effect on vascular dementia [22]. We detected the content of nitric oxide (NO), malondialdehyde (MDA), reactive oxygen species (ROS) and the activity of superoxide dismutase (SOD) in the hippocampus of rats (Fig. 4). It was found that oxidative stress injury in the hippocampus of rats induced by VD. Compared with the normal saline group, the content of NO decreased, the content of MDA increased, the content of ROS increased and the activity of SOD decreased in VD rats. After the intervention of PAE, this phenomenon can be significantly reversed. Moreover, the content of MDA was the lowest and the content of SOD was the highest in the PAE + mNGF group, which indicated that the PAE + mNGF group had the foremost antioxidant effect among all the therapeutic groups.

### PAE significantly inhibited apoptosis of hippocampal neurons in VD rats

**Fig. 5:**
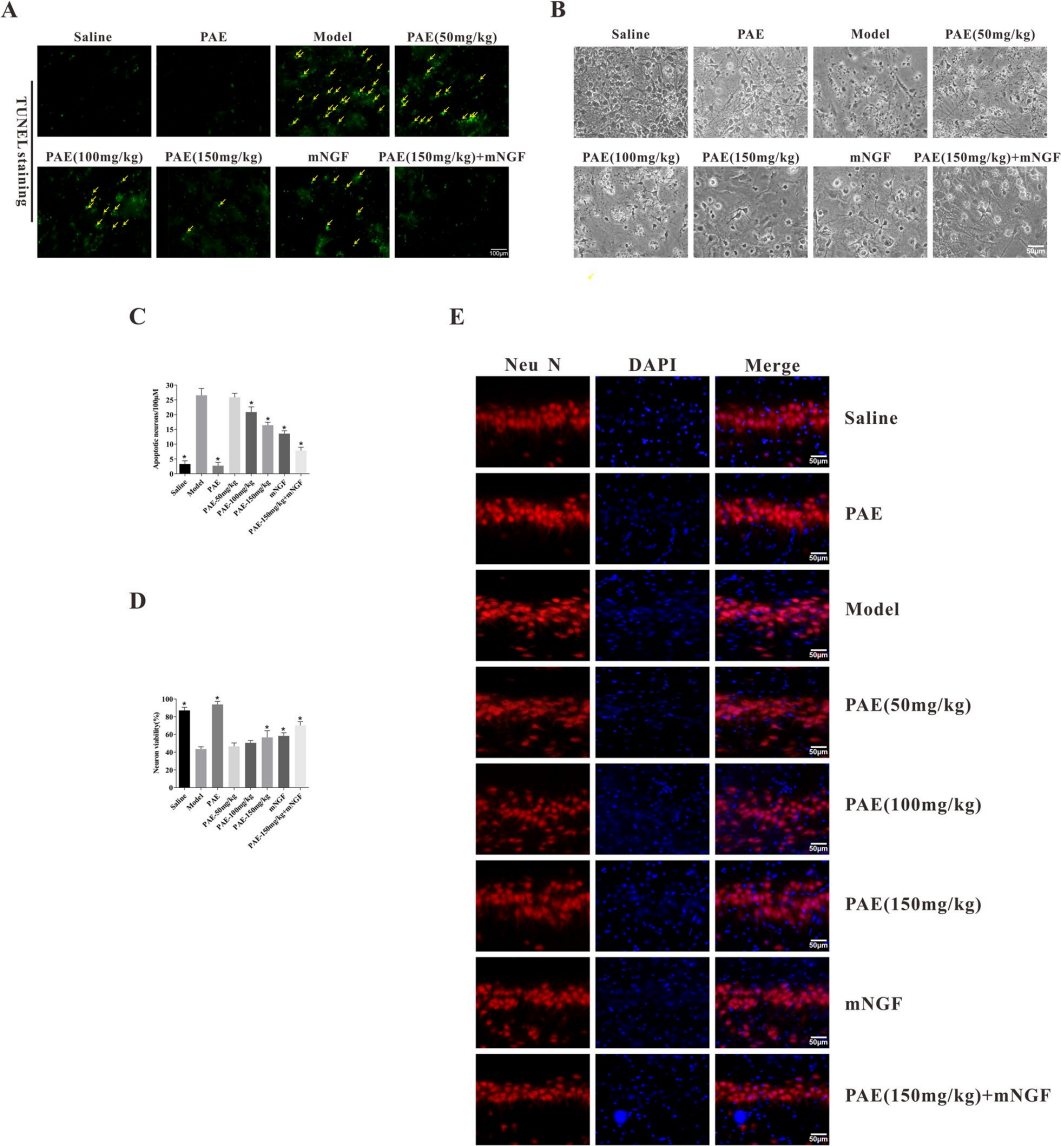
PAE inhibited the apoptosis of hippocampal neurons in VD rats. **A** Representative photos of TUNEL staining in each group (×400). **B** Quantitative analysis of the normal neuron and **C** apoptotic cells. **D** Quantitation of damaged neurons after PAE treatment by MTT assay. Neuronal viability was significantly increased after PAE treatment. **E** Representative image of Immunofluorescence staining for Neu N (red) in the hippocampus from different groups. All data is expressed as mean ± SEM. N is 6 in each group. *P < 0.05 vs. Model group

TUNEL staining was performed to detect the effect of PAE on apoptosis of hippocampal neurons. There were lots of TUNEL positive cells in the model group, showing the unique morphological characteristics of apoptotic cells. The number of TUNEL positive cells in the hippocampus of the PAE-treated rats was significantly reduced compared with the VD model rats (Fig. 5A, C). Further, it was observed under light microscope that the cell body of primary hippocampal neurons in sham operation group was conical, with strong refraction sensation, obvious axons and dendrites, and dense terminal network. The processes of primary hippocampal neurons in VD rats were retracted or broken, the cell body shrinkage was round and dotted, and the network disappeared (Fig. 5B). MTT staining verified a significant increase in neuronal viability following PAE treatment (Fig. 5D). Neu N immunoreactive neurons are mainly distributed in the granule cell layer (GCL) and stratum pyramidale (SP). NeuN fluorescence staining was used to assess the loss of synapses and neurons in hippocampus from different groups. As shown in Fig. 5E, the morphology and structure of neurons in the hippocampus of rats were significantly improved after PAE treatment, especially the high-dose PAE treatment could significantly reverse the damage of hippocampal neurons in VD rats. At the same time, high-dose PAE and mNGF could significantly improve the morphological structure of hippocampal neurons.

### PAE inhibited the expression of TRPM2 downregulating the abnormal increase of cytoplasmic Ca^2+^ via NMDAR pathway in hippocampal neurons

**Fig. 6:**
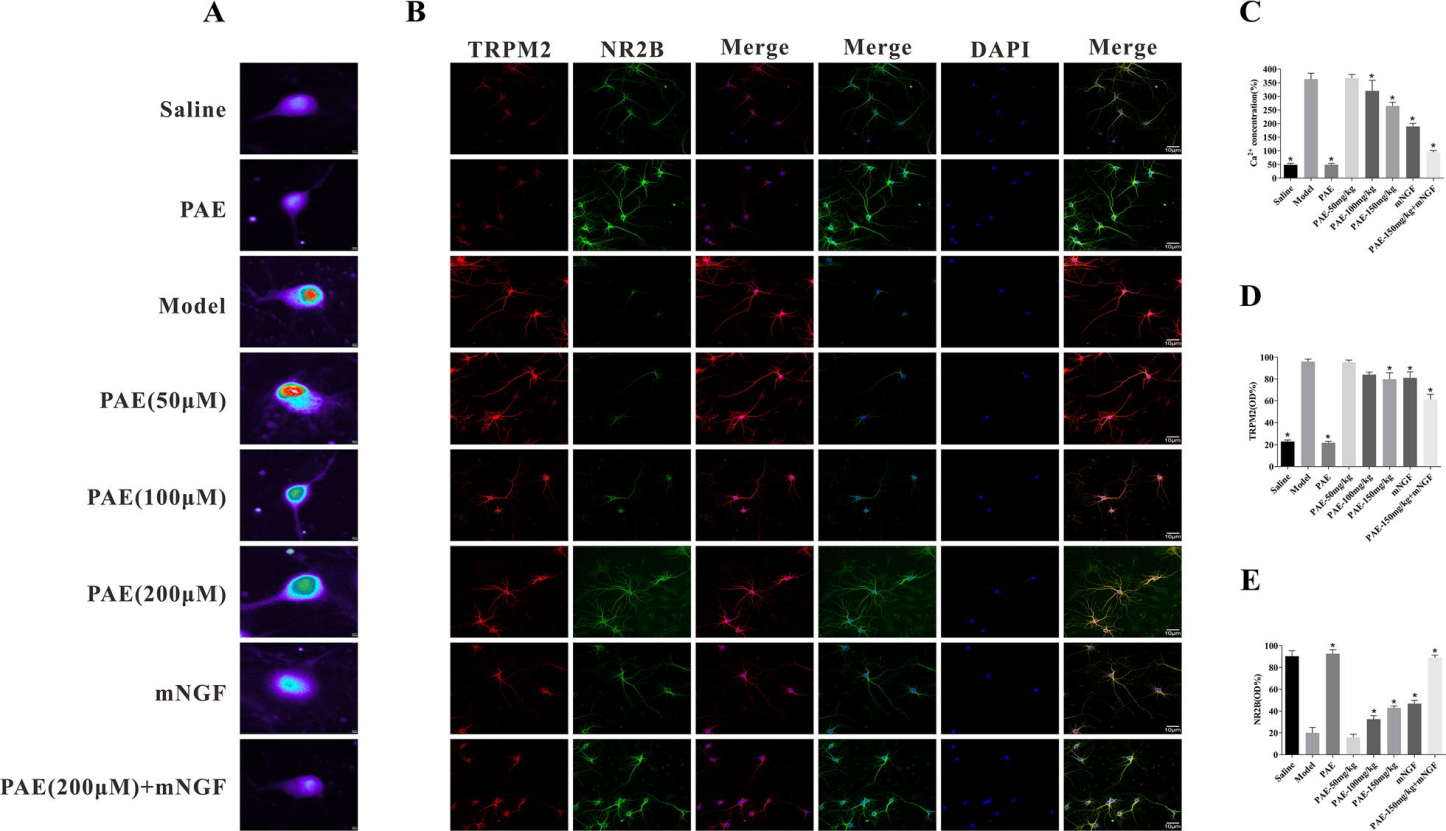
PAE upregulated the protein expression of NR2B and downregulated the protein expression of TRPM2. **A** Fluo-3/AM was used to measure the cytosolic calcium concentration in hippocampal neurons (×800). **B** Immunofluorescence detection of NR2B and TRPM2 in hippocampal neurons (×200). **C** Quantitation of cytoplasmic Ca^2+^ fluorescence. **D** Quantitation of TRPM2 fluorescence expression intensity. **E** Quantitation of NR2B fluorescence expression intensity. All data is expressed as mean ± SEM. N is 6 in each group. *P < 0.05 vs. Model group

**Fig. 7:**
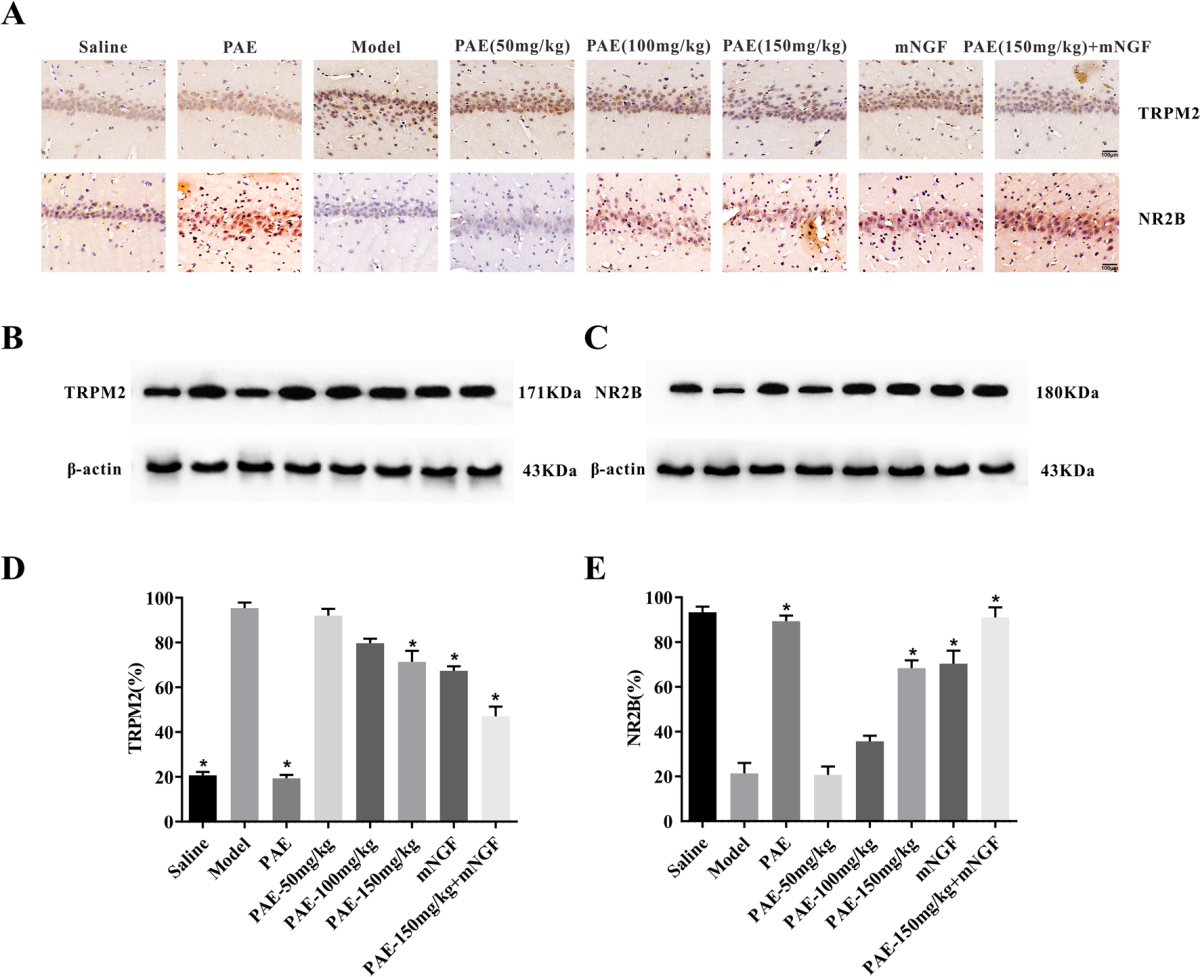
Expression levels of NR2B and TRPM2 in the hippocampus tissue. **A** Immunohistochemistry of NR2B and TRPM2 in the hippocampus tissue (×200). **B**, **C** Representative Western blotting of NR2B and TRPM2 in the hippocampus tissue. β-Actin was used as the loading control. **D**, **E** Corresponding histograms. All data is expressed as mean ± SEM of at least three independent experiments. *P < 0.05 vs. Model group

Ischemic neuronal death is caused by the activation of glutamate and glutamate-independent channels, resulting in intracellular calcium overload. The traditional mechanism of glutamate receptor-driven excitotoxicity is caused by the overactivation of *N*-methyl-d-aspartate glutamate receptor (NMDARs). Some studies have shown that the down-regulation of TRPM2 has a neuroprotective effect, which is related to the mechanism of NMDAR regulating calcium concentration [23]. At the cellular level, we found that the fluorescence intensity of cytoplasmic Ca^2+^ in the model group was significantly higher than that in the normal saline group, and PAE could antagonize the increase of cytoplasmic Ca^2+^ fluorescence intensity induced by the model group (Fig. 6A). Thus, we detected whether PAE affects TRPM2 and NR2B protein expressions in vivo. Double immunofluorescence (IFC) assay was used to detect the expression of TRPM2 and NR2B in hippocampal neurons (Fig. 6A, C). The results showed that the model group dramatically decreased the protein levels of NR2B but increased TRPM2, compared with saline-treated cells. After PAE-treated, these phenotypes were reversed, especially in the high-dose group (Fig. 6B, D, E). This effect was obviously enhanced when PAE and mNGF intervened together. We next determined the protein levels of TRPM2 and NR2B in hippocampal tissues by IHC (Fig. 7A). The results showed that the expression trend of TRPM2 and NR2B was consistent with that of cell level. Similarly, the results of western blotting showed that the levels of TRPM2 were increased and NR2B were decreased in VD rats, which were reversed by PAE and mNGF treatments (Fig. 7B, C), further supporting the notion that PAE is a considerable drug to prevent VD.

## Discussion

The major finding in this project is that PAE can ameliorate functional impairment in rats modeling vascular dementia in a dose-dependent manner. Our results demonstrated that PAE improved the morphology and structure of hippocampal neurons and rescue dendritic remodeling in the hippocampus and also improved spatial learning and memory. PAE increased the activity of superoxide dismutase and decreased the formation of MDA, thus inhibited oxidative stress in neurons. PAE also inhibited TRMP2 and upregulate NR2B levels to downregulate the abnormal increase of cytoplasmic Ca^2+^ in the hippocampus.

Vascular dementia (VD) is one of the most common types of senile dementia, second only to Alzheimer’s disease (AD). With the increase of social aging, VD has become an important event hindering the quality of life of patients, which has brought great spiritual and economic burden to society. However, the pathogenesis of VD is still unclear. In addition to cardiovascular risk factors, it also involves white matter damage, apoptosis, cytokines, excitatory amino acid toxicity, free radical damage and so on. PAE is a promising monoterpenoid extracted from *perilla*, with a variety of biological activities, such as anti-inflammatory, antibacterial, antidepressant, anti-tumor, sedative and so on [24, 25]. It was vital that PAE does not pose any risk to the human body and can be used as a daily flavoring for inflammatory diseases [26, 27].

Pathological studies show that oxidative stress and inflammatory markers exist in the damaged white matter associated with vascular cognitive impairment (VCI) [1]. Appropriate supplement of neurotrophic factors and reduction of inflammatory factors can show a modest cognitive improvement [22]. Our results show that PAE can significantly reduce the expression levels of MDA and ROS in the brain tissue of VD rats, and significantly increase the level of SOD. At the same time, the number of neurons in the brain tissue is also significantly increased. These results suggest that PAE can prevent the decrease of endogenous antioxidant enzyme activity, and may prevent the loss of neuron number after ischemia–reperfusion injury through antioxidant pathway, thus providing neuroprotective effect. Therefore, we suggest that PAE can improve cognitive impairment induced by VD through reducing oxidative stress and other mechanisms.

mNGF is implicated in nourishing, preserving and promoting the survival and growth of synapses [28]. Some studies showed that the pathogenesis of Alzheimer’s disease (AD) and vascular dementia (VD) was related to the lack of mNGF protection [29]. Therefore, increasing the content of mNGF in the nervous system is one of the effective ways to reduce the damage of nervous system after cerebral ischemia. This protective mechanism involved mNGF preventing apoptosis, activating the signal transduction pathway of myoinositol triphosphatase, promoting the survival of neurons, keeping the level of intracellular free calcium stable, and protecting neurons from ischemic injury [30]. Here, the results showed that the appropriate supplement of mNGF had a protective effect in vascular dementia rats, and mNGF combined with PAE showed a better therapeutic effect, suggesting that the mechanism of PAE may also involve the regulation of calcium channels.

In ischemic cell death, TRPM2 is activated by oxidative stress products (ADPR and H_2_O_2_), which leads to calcium overload [31]. The increase of intracellular calcium level leads to mitochondrial dysfunction and ROS production, which further activate glutamate and nonglutamate calcium channels [32]. However, inhibition of TRPM2 expression by antagonists or gene silencing could inhibit calcium influx induced by ADPR and H_2_O_2_ [31–33]. Alim et al. Confirmed that the increase of fEPSPs excitability in TRPM2^−/−^ mice depended on the up regulation and down regulation of NR2BA and NR2BB, which ultimately reduced the vulnerability of TRPM2^−/−^ mice to cerebral ischemia [17]. In this study, we found that the down regulation of TRPM2 and up regulation of NR2B regulated by PAE can significantly reduce the calcium concentration in the injured neurons and improve the cognitive impairment of vascular dementia rats.

A major limitation of this study is that we did not reveal the mechanism underlying how PAE decreases TRPM2 and increases NR2B protein levels to prevent cognitive impairment of vascular dementia rats. TRPM2 was first proposed by Alim as a switch to regulate the ratio of NR2BA and NR2BB to affect the ischemic cell death. NMDAR signaling pathway not only regulates synaptic calcium influx, but also regulates downstream activation of MEK and PI3 kinases, which are necessary for ERK1/2 and Akt phosphorylation, respectively. Akt phosphorylation further inhibited the expression of proapoptotic factor GSK3β [17]. ERK1/2 also mediated microglial phenotype and increased pro-inflammatory response. Prajwal provided a unified mechanism to link elevated glutamate levels with chronic neuroinflammation, including PARP-1/TRPM2 mediated the pro-inflammatory effect of NMDAR stimulation. ERK1/2 phosphorylation activated PARP-1, which generated polymers of ADPR further activating TRPM2 channel [34]. Here, we have confirmed that PAE intervention can trigger NMDAR/TRPM2 pathway leading to significantly reducing the apoptosis of hippocampal neurons (Fig. 5A). Next, we plan to further elucidate the molecular mechanism of PAE in improving vascular dementia rats from the relationship between NMDAR/TRPM2 signaling pathway and pro-inflammatory response of microglia.

## Conclusion

Overall, this study provides a novel function of PAE which inhibits TRPM2 and increases NR2B to reduce the calcium overload to prevent cognitive dysfunction. As a result, PAE attenuated the neuron damage in vascular dementia rats. These findings suggest that PAE can be used as a protective strategy for improving the function of cerebrovascular dementia.

## Data Availability

The data used to support the current study are available from the corresponding author on reasonable request.

## Abbreviations

PAE: Perillaldehyde
VD: Vascular dementia
NR2B: *N*-Methyl-d-asprtate receptor-2B
TRPM2: Transient Receptor Potential Melastatin 2
NeuN: Neuron-specific nuclear protein
TUNEL: TdT-mediated dUTP nick end labeling
No: Nitriteoxide
MDA: Malondialdehyde
SOD: Superoxide dismutase
ELISA: Enzyme linked immunosorbent assay
